# A survey study to validate a four phases development model for integrated care in the Netherlands

**DOI:** 10.1186/1472-6963-13-214

**Published:** 2013-06-13

**Authors:** Mirella MN Minkman, Robbert P Vermeulen, Kees TB Ahaus, Robbert Huijsman

**Affiliations:** 1 Vilans, National Center of Excellence for Long-term care, PO Box 8228, 3503, RE Utrecht, The Netherlands; 2 Thorax Center, University Medical Center Groningen, University of Groningen, PO Box 30.001, 9700, RB Groningen, The Netherlands; 3 Faculty of Economics and Business, Research Center on Healthcare Organization & Innovation. University Medical Center Groningen, University of Groningen, Landleven 5, 9747, AD Groningen, The Netherlands; 4 Institute of Health Policy and Management, Erasmus University Rotterdam, PO Box 1738, 3000, DR Rotterdam, The Netherlands

**Keywords:** Integrated Care, Development Phases, Development Model for Integrated Care

## Abstract

**Background:**

The development of integrated care is a complex and long term process. Previous research shows that this development process can be characterised by four phases: the initiative and design phase; the experimental and execution phase; the expansion and monitoring phase and the consolidation and transformation phase. In this article these four phases of the Development Model for Integrated Care (DMIC) are validated in practice for stroke services, acute myocardial infarct (AMI) services and dementia services in the Netherlands.

**Methods:**

Based on the pre-study about the DMIC, a survey was developed for integrated care coordinators. In total 32 stroke, 9 AMI and 43 dementia services in the Netherlands participated (response 83%). Data were collected on integrated care characteristics, planned and implemented integrated care elements, recognition of the DMIC phases and factors that influence development. Data analysis was done by descriptive statistics, Kappa tests and Pearson’s correlation tests.

**Results:**

All services positioned their practice in one of the four phases and confirmed the phase descriptions. Of them 93% confirmed to have completed the previous phase. The percentage of implemented elements increased for every further development phase; the percentage of planned elements decreased for every further development phase. Pearson’s correlation was .394 between implemented relevant elements and self-assessed phase, and up to .923 with the calculated phases (p < .001). Elements corresponding to the earlier phases of the model were on average older. Although the integrated care services differed on multiple characteristics, the DMIC phases were confirmed.

**Conclusions:**

Integrated care development is characterised by a changing focus over time, often starting with a large amount of plans which decrease over time when progress on implementation has been made. More awareness of this phase-wise development of integrated care, could facilitate integrated care coordinators and others to evaluate their integrated care practices and guide further development. The four phases model has the potential to serve as a generic quality management tool for multiple integrated care practices.

## Background

### Integrated care development

Numerous studies of integrated care define and discuss the interventions that need to be implemented in order to streamline care processes and organise collaboration between professionals and organisations [1-5]. Integrated care can be defined as a coherent and coordinated set of services, which are planned, managed and delivered to individual service-users across a range of organisations and by a range of cooperating professionals and informal care-givers [2]. Whereas the rationale for integrated care is evident, the developmental process for integrated care is less clear as it is a complex and long-term one. The integration of care can be complicated by different goals, different funding streams and different stakeholders or care providers.

To research the development of integrated care, it is interesting to involve literature of related subjects like networks and the development of organisations. In the literature about the development of organisations, numerous authors have described life cycle models, often with three to five phases [6-9]. Major organisational problems like for example increased competition or budget cuts can generate the necessary urgency and activity for further development, resulting in another phase of the life cycle. Although there is no consensus in the literature about the number of phases and the phase definitions, there is a consensus that organisations change over time in order to survive. Moreover, the assumption that organizations do experience life cycles is based on literature that is mainly conceptual and descriptive in nature. The parallel with linear growth stages is doubted, and an evolutional or discontinuous perspective appears more realistic.

The development of networks is another related area. A network is defined as a more or less stable pattern of social relations among different actors (people, groups or organisations). Network development is characterised by continuous restructuring and reshaping as a result of the actions, interactions and interpretations of the parties involved [5]. Because integrated care concerns health care organisations and their collaboration in differing degrees of intensity and in different appearances, these perspectives about organisational and network development are useful when researching the development of integrated care.

### A four-phase development model

In a previous study [10,11] we developed a four-phase model for integrated care services (see also methods), namely the Development Model for Integrated Care [DMIC see Table 1]. We performed a literature search on integrated care development and the findings were used by an expert panel to build the DMIC. The expert panel reached consensus that different phases of development can be identified in integrated care practices. For instance, some stroke services are already measuring the results of the care process and have reached agreements with the care providers involved, whereas others are still starting up the collaboration. The experts stressed that thinking in terms of phase-wise development is relatively new and that this is therefore still scarcely used by integrated care practitioners. Besides the four phases, the model consists of 89 elements of integrated care grouped in nine clusters. An element was defined as an activity focusing on the development (realisation, improvement, innovation of sustainability) of integrated care [10,11].

**Table 1:** The development phases of integrated care

**PHASE 1 Initiative and design phase**	The collaboration between health care providers has been intensified or started up. The starting point is a common problem or chance occurrence, or builds on current cooperation among care professionals. There is a sense of urgency and there are possibilities for working on these challenges in collaboration. The targeted patient group, the care chain and care process have been defined, as also the needs of patients and stakeholders. The level of ambitions, motivation and leadership determine the progress achieved. A multidisciplinary team designs an experiment or project to execute the current ideas. The collaboration can be signed up to in an agreement among care partners.
	*Key words*: Exploring possibilities/impossibilities, ambitions and chances, (project) design and collaboration agreements.
**PHASE 2 Experimental and execution phase**	New initiatives or projects are being executed in the care chain. The aims, content, roles, and tasks in the care chain have been clarified and written down in care pathways and protocols. There is coordination at the level of the care chain by for instance installing coordinators or setting up meetings. Information about patient groups, working procedures or professional knowledge is exchanged. There are experiments within the collaboration, results are evaluated to learn from and reflect on. Preconditions for projects have been considered and boundary conditions have been solved by collaborative means or agreements among care providers.
	*Key words*: Writing down aims and content of the collaboration, coordination at care chain level, experimenting and reflecting.
**PHASE 3 Expansion and monitoring phase**	Projects have been expanded or integrated in integrated care programmes. Agreements on the content, tasks and roles within the care chain are clear and signed up. Collaboration is no longer on an informal basis. Results are systematically monitored and improvement areas identified. The targeted population has been surveyed. More collaborative initiatives emerge such as mutual education programmes. There is a continuous commitment to the ambition of the integrated care programme. Interorganisational barriers and fragmented financial structures are on the agenda of the care partners.
	*Key words*: Further development and maturity, monitoring and improving results, new questions and innovation.
**PHASE 4 Consolidation and transformation phase**	The integrated care programme is the regular way of working and providing care. Coordination at care chain level is operational; information is shared, transferred and fed back. A monitoring system periodically shows if results are being sustained, what specific improvement possibilities have been identified and to what extent patient needs have been met. The programme builds further on successful results. Organisational structures transform or are newly designed around the integrated care programme. Financial agreements are arranged with financers by means of integral contracts covering the care chain as a whole. Partners in the care chain explore new options for collaboration in the external environment with other partners.
	*Key words*: Continuous improvement, new ambitions, structures fitting the integrated care programme (organisational structures, integral financing).

In this model, the first phase was labelled the initiative and design phase, where a new chance occurrence initiated a new cooperative arrangement or a current arrangement is intensified. The care process and client group are defined. In the second experimental and execution phase, improvement plans and care pathways are implemented, and coordination mechanisms are arranged. In the third expansion and monitoring phase, roles and tasks have become clear and are formalised. The target population is monitored as well as the results of the integrated care. Once the integrated care programme has become the regular way of working, organisational structures are in process of transformation and integrated financial budgets have become a topic of discussion, the fourth consolidation and transformation phase has been reached (see Table 1).

Although the phase descriptions were developed in a structured way by the expert panel [11,12], they have not yet been validated in practice. Our aim in this study was to assess if the earlier literature- and expert-based developed phases of integrated care are also confirmed empirically in practice in the Netherlands. For this empirical validation we selected three essentially different types of integrated care services: for patients with stroke, acute myocardial infarction (AMI) or dementia.

To assess whether the development phases could be confirmed, we used a four step approach with multiple research questions.

1. Are the conceptual development phases recognised in integrated care practice? Are previous phases also recognised?

2. Is there a relationship between the development phases and (a) the number of implemented elements of integrated care or (b) the number of planned elements of integrated care?

3. Are elements corresponding to earlier phases also earlier implemented in practice?

4. What factors are crucial for moving to the next phase of development?

5. Further, we were also interested in the resemblance or differences between self-assessed development phase scores of the respondents, and the development phases as calculated by the model.

6. Development of integrated stroke, AMI and dementia care in the Netherlands.

To validate the four-phase model and to assess its generalizability, we researched three groups of integrated care services that vary on both quantitative as qualitative characteristics. Each year 41,000 people suffer from stroke, 36,000 from AMI and as many as 230,000 people are diagnosed with dementia in the Netherlands (out of 16.7 million inhabitants, of whom 15.6% are 65 or over) and these numbers will increase in the near future [13-15]. All three integrated care services are being developed among general practitioners, hospital care and ambulatory services. For stroke and AMI patients, acute care services play an important role. In the case of stroke services, rehabilitation centres, rehabilitation wards in nursing homes or home care organisations provide care after the patients are discharged from hospital. In dementia care mental health services, social care and informal services also play an important role. In the Netherlands integrated care services often are supported by an integrated care coordinator or project leader, which operates on a tactical level in the collaborative network. Integrated care coordinators are responsible for supporting the process of planning and improving the integrated care service, improving collaboration and monitoring the results. There are not only differences between these three groups of integrated care services, but within the groups regional characteristics such as the presence of providers and facilities (for instance a rehabilitation clinic) and earlier collaboration also influence the members involved in the service.

Another difference between these three patient groups is evident from their development history. Stroke services were one of the first integrated care services in the Netherlands and were first started up in the late 1990s. They are defined as a network of service-providers working together in an organised way to provide adequate services at all stages of the follow-up care for stroke patients [16]. Stroke services worked on improving patient flows from hospitals to nursing homes and on improving information flows and the implementation of thrombolysis for acute ischaemic stroke patients [17-19]. Later on, care in the chronic phase and involving patients as partners became a focus. The development of AMI services started some years later and focused on arranging primary percutaneous coronary intervention (PCI) in a timely fashion, making agreements concerning pre-hospital diagnosis, arranging direct transfers to catheterisation laboratories (bypassing general hospitals and emergency departments), and post-intervention patient management. Nowadays they are working towards a better understanding of the role of each health care-provider as part of the integrated care service, and are implementing continuous self-monitoring and improvement strategies [20]. The development of dementia care started only about five years ago in response to policy-makers and client federations who were concerned about the fragmentation of dementia care. National initiatives like the National Dementia Programme and the Front Runner Programme were started up and focused on a more active role for the general practitioner, more diagnostics and better and coherent care after diagnostics for both the patient and their care-givers. Other topics were the implementation of extensive case management, client and family involvement, a national dementia indicator set and the development of a method for financial agreements between providers and insurers in a specific region [21,22].

## Methods

### Pre-study

In a pre-study we constructed the Development Model for Integrated Care (DMIC) by means of a literature study, a three-round Delphi study with 31 experts, a Concept Mapping study and an additional questionnaire research [10-12]. The four development phases (Table 1), together with nine clusters and 89 elements, are components of the model. During a session with 27 highly qualified experts on integrated care, the experts reached consensus that integrated care development could be described in four phases. An additional questionnaire survey was performed (among 29 experts) to assess if the concept phase descriptions were recognised in practice by the experts. Analyses of the results showed a high confirmation of the phase descriptions. Only one expert did not recognise one phase. The experts reviewed all of the 89 elements and scored in which phase elements were ‘relevant’ (in one or multiple phases) and were ‘mostly relevant’ (in one of the four phases). Based on these expert scores, lists of the elements that are most related to each phase were constructed [11].

### Questionnaire survey on integrated care services

For this evaluative survey study we compiled a three-part Excel-based questionnaire (A-C). Part A focussed on general information about the integrated care service. Data were collected on the starting year, the number of patients covered in the previous calendar year, the number and type of health care providers involved, current agreements between care providers, the infrastructure for improvement, the availability of a coordinator at care chain level and commitment at strategic level (12 items). In part B the respondents rated the 89 elements of the DMIC in terms of relevance and existence in daily practice and where applicable since which year (89 items). In part C the descriptions of the four development phases were presented and the respondents each assessed their own development phases. Further questions concerned the completion of previous development phases, the duration of phases and the crucial factors for moving onto the next phase (6 items). Filling in the questionnaire took about one hour.

### Integrated care settings

Coordinators of integrated stroke, AMI and dementia services were invited to complete the questionnaire. The rationale for our research in these groups was based on the aim of variety in patient groups covering a range from acute to chronic care. The service varied in terms of the providers involved, sectors and years of development, so that different integrated care settings in different stages of development were included. A criterion was the availability of an integrated care coordinator at a tactical level, also sometimes called an integrated care director, project or programme leader. The coordinator had to have a good overview of the current state, history and future plans of the total integrated care service. The coordinator was in the lead to fill in the questionnaire (one per service) and was allowed to consult partners. Another criterion was the availability of a national collaborative network with a good geographical spread. The National Stroke Service Network, the National Network on Dementia and the National Society for Trauma Centres are such networks and they all wrote to their members recommending participation. All 36 stroke services, 50 dementia care services and 12 myocardial services in the Netherlands were invited to participate.

### Respondents

For each service we selected the coordinator at a tactical level as a single respondent. Respondents were allowed to contact other partners in their integrated care setting to provide input for the questionnaire. The coordinator was contacted by phone or e-mail. After accepting our invitation, the Excel-based questionnaire and an instruction sheet were e-mailed to the respondent. Due to the smaller numbers, the participating AMI services were visited by one of the researchers to introduce them to the questionnaire and assess the right respondent. Non-responders were reminded twice by telephone or by e-mail. If available, the reasons for non-response and remarks on the questionnaire were documented. For this study, no ethical approval was needed. The collected data did not address any individual nor group wise patient data. The focus was on organisational aspects of integrated care (development phases) which were delivered on a voluntary basis by the integrated care coordinators.

### Analyses

Descriptive statistics were used to analyse the first and last part of the questionnaire (case characteristics, question 1). To analyse the second and third research question we further used descriptive statistics, Kappa and Pearson’s correlation tests. To analyse the fourth research question, open questions were formulated in the questionnaire. We compared the content of the answers of the respondents (crucial factors for development) with the content of the set of 89 elements [10] and scored which elements and how often they were named. We used descriptive statistics. Data were analysed by using SPSS software version 16.0.

To analyse if the self-assessment scores for each integrated care service corresponded with what was expected by the model, we also calculated phase scores for each service, based on the number of relevant and implemented elements and the overlap with the top-ten elements per phase made by the experts [11]. The top-ten elements can be considered as a set of elements that is the most related to and representative for that phase. We considered multiple methods to identify the phase of integrated care development. These were: (a) to regard a phase as completed if 6, 7, 8, 9 or 10 out of 10 elements in the corresponding phase had been implemented; and (b) to divide the total number of implemented elements out of a possible 40 by ten, and rounding to the nearest integer. The number thus obtained corresponded with the current phase of development. For all these methods, we used Kappa tests to study the correlation between self-assessed and calculated phases.

## Results

### Response and characteristics

The overall response rate to the questionnaire was 83%; 32 out of the 36 stroke services (89%), 9 out of the 12 AMI services (75%) and 43 out of the 50 dementia services (86%) participated. Reasons for non-response were lack of time or absence because of illness or holidays. The main characteristics of the integrated care services that participated are presented in Table 2. The table shows a variation between the three groups in, for instance, the average start year, the number of clients and the care providers involved. For dementia no central databases with total client numbers were available. The percentage with an integrated care coordinator ranged between 33% (AMI) and 96% (dementia). The services with not a formal coordinator were contacted or visited (AMI) to assess which person(s) with coordinative tasks (for instance the project leader) best could fill in the questionnaire. The designated time available to each coordinator ranged from two to 15 hours on average per week, with a median of 8.5 hours. Because the coordinator had an important role in our study by representing the integrated care service, we analyse whether the available coordination time was related to the overlap in self-assessed phase scores and calculated phases. For this purpose, we divided the group coordinators into two, based on whether the number of dedicated coordination hours was more or less than the median. The Kappa scores of the group of coordinators with more coordination time (≥ 8.5 hours/week) were slightly higher for four out of the seven calculation methods compared to the group with limited time (≤ 8 hours/week), and could not be computed for the 9 and 10 out of ten rule. This indicates a possible higher identification with the phases as designed in the model, when coordinators have more time to spend on their integrated care coordination.

**Table 2:** Characteristics of participating integrated care services

**Characteristic**	**Stroke n** = **32**	**AMI n** = **9**	**Dementia n** = **43**
*Average start year* (*min* – *max*)	*2001* (*1996*–*2005*)	*2003* (*1998*–*2004*)	*2007* (*2000*–*2010*)
*Average age in years* (*sd*)	*7*.*75* ± *2*.*4*	*5*.*67* ± .*2*.*0*	*2*.*72* ± *2*.*1*
*No*. *of patients in 2008*	*449* ± *340*	*1109* ± *515*	*Nd*
(*min* – *max*)	(*134* – *1914*)	(*519* – *2200*)	
*Care providers involved* (% *of n*):			
- *hospitals*	*100*	*100*	*91*
- *expertise centre*	*nd*	*nd*	*47*
- *mental health care*	*0*	*0*	*98*
- *rehabilitation centre*	*88*	*0*	*0*
- *nursing and elderly homes*	*100*	*11*	*100*
- *home care*	*100*	*0*	*100*
- *welfare*/*social care*	*nd*	*0*	*77*
- *client organisation*	*38*	*0*	*98*
- *municipality*	*13*	*0*	*72*
*Agreements available with*: (% *of n*)			
- *general practitioners*	*72*	*89*	*56*
- *ambulances*	*78*	*100*	*0*
% *with integrated care coordinator*	*78*	*33*	*96*
*Average hours per week* (*min*-*max*)	*5*.*5* (*0*–*19*)	*2*.*0* (*1*.*5*-*2*.*4*)	*15* (*2*–*36*)
% *with improvement teams at care chain level*, *consisting of*	*91*	*78*	*91*
- *professionals*	*3*	*100*	*13*
- *managers*	*3*	*0*	*3*
- *mixed*	*93*	*0*	*85*
% *with formal collaboration agreement between involved providers*	*69*	*22*	*84*
% *with regular board meetings of involved providers*	*78*	*67*	*95*
% *with periodically meetings with*:			
- *health insurers*	*19*	*11*	*28*
- *care administration offices*	*28*	*11*	*93*
- *care assessment organisations*	*25*	*0*	*14*

Nd = no data available.

### Recognition of the phases of development and previous phase (question 1)

**Figure 1:**
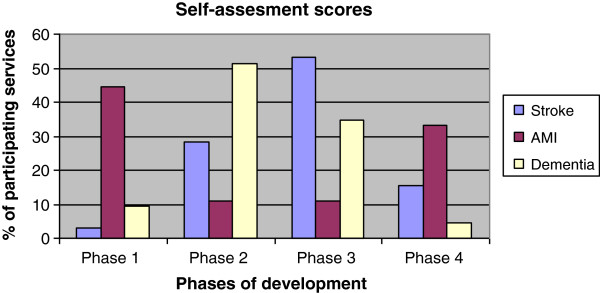
**Self**-**assessed phase for three types of services.**

All integrated care services self-assessed their development phase (Figure 1). Overall, the respondents felt able to position their practice in one of the four described phases. Some respondents commented that elements from later phases were also recognised in the current phase or remarked that their integrated care was about to enter the next phase. For stroke, one integrated care service self-scored their practice in phase one; the most self-scored phases were in phase three (n = 17) and two (n = 9). The AMI services most self-assessed phase one (n = 4) and four (n = 3). The dementia services covered all phases, with the most self-assessment scores in phase two (n = 22) and three (n = 15). The service coordinators who self-assessed a phase two to four were asked if they had been through the previous phase as presented in the description. Of the respondents 92% (n = 75 with assessed phase 2 to 4, 4 missing) confirmed that they recognised and had completed the previous phase.

### Relationship between phases and implemented and planned elements (question 2)

**Figure 2:**
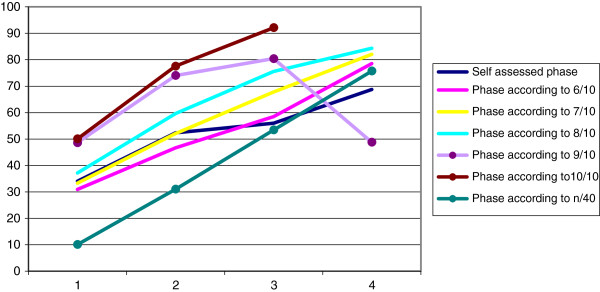
**Percentage of implemented**, **relevant elements per phase.**

To assess whether services in further phases of development have taken more steps towards realising integrated care, we calculated the number of implemented elements that were considered relevant. Figure 2 shows the mean percentages of relevant *implemented* elements per phase, stratified by the self-assessed phase and the calculated phases according to the calculation methods. For all methods, the number of elements implemented on average increases over the phases. For the self-reported phase, correlation with number of relevant items implemented was lowest (Pearson’s R 0.397). For the calculation methods, Pearson’s R was up to ≥ 0.9 (for the 6/7-10 and number of implemented out of 40/10 method). The average number for the total group was 46 ± 20 elements (range 3–82). For the three subgroups, 50 ± 18 (10–77) elements for stroke, 42 ± 13 (20–61) elements for AMI and 45 ± 22 (3–82) elements for dementia were implemented.

**Figure 3:**
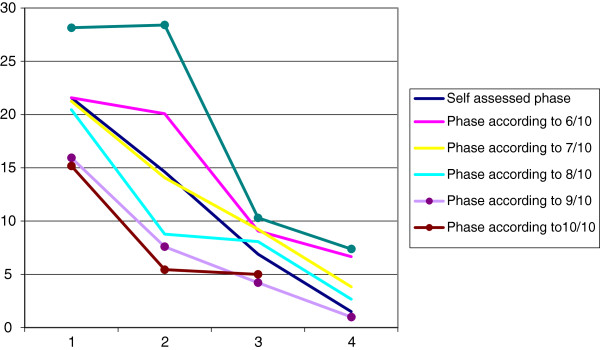
Percentage of planned, relevant elements per phase.

The average number of *planned* elements for the total group was 14 ± 14.6 (min 0, max 57). Figure 3 shows the mean percentages of relevant planned elements, stratified by the self-assessed and calculated phases according to the calculation methods. For all methods, the number of elements planned on average decreased over the phases. We also analysed for each calculation method the mean number of planned elements that belong to the current phase plus one. In other words, we looked for those elements of our model that mark the transition from the current to the next phase. We found that there was no relation between current phases and planned elements belonging to the next phase, indicating that although plans are being made for development of the care service, these are not necessarily aimed at the next stage of collaboration.

### Are elements belonging to earlier phases also earlier implemented? (question 3)

To assess if elements of earlier phases were also implemented at an earlier moment, we analysed the age of the top-ten elements of all phases for the three groups [11]. Table 3 shows that implemented elements for stroke and AMI in phase 1 and 2 are ‘older’ (e.g. implemented earlier) than elements of phase 3 and 4. This distinction is absent for dementia services, which are younger than the other two services.

**Table 3:** Mean age in years of elements in different phases

**Type**	**Stroke (n** **=** **32)**	**AMI (n** **=** **9)**	**Dementia (n** **=** **43)**
Average age of phase 1 elements (sd)	6.8 (2.5)	5.2 (2.6)	1.9 (1.8)
Average age of phase 2 elements (sd)	6.7 (2.0)	5.5 (1.6)	2.2 (2.2)
Average age of phase 3 elements (sd)	6.1 (2.1)	4.6 (1.7)	2.3 (2.2)
Average age of phase 4 elements (sd)	5.7 (2.5)	4.4 (2.3)	1.8 (1.5)

### Crucial factors for developing to a next phase (question 4)

Analyses of the qualitative data on the crucial factors for moving on to a next phase (based on self-assessed scores) showed differences and some similarities between phases (see Table 4). For all phase transitions, the commitment of CEOs and the higher management levels of the participating organisations was most frequently mentioned (n = 24). Also financial agreements between involved parties and financial preconditions for realising the integrated care and improvement activities were mentioned for all phase transitions (n = 16). For the transition from the first to the second phase, the installation of a coordinator, agreements about tasks and responsibilities, care-pathways and case management, improvement teams and the commitment to the defined ambitions and aims were named (all 3 times). To transform to the third phase of development, installing a coordinator, agreements about tasks and responsibilities, room for experiments and an open environment, and adjustments between care professionals by direct contact were most frequently mentioned. Fewer elements were mentioned concerning the transition to the fourth phase. Only CEO and higher management commitment and financial agreements between care partners were mentioned by multiple services (n = 3). Factors that were mentioned which were not included as an element in the 89 elements of the model [10] were participation in a national improvement programme (n = 5), sustainability activities after a project phase (n = 3), external pressure (by the ministry or healthcare insurers) and equity in the relationship between care providers (both n = 2).

**Table 4:** Crucial elements for phase transitions

**Element**	**N** **=** **30**	**N** **=** **30**	**N** **=** **8**
	**1-** **>** **2**	**2-** **>** **3**	**3 -** **>** **4**
Assuring the leadership commitment of the partners involved in the care chain	++	++	+
Allocating financial budgets for the implementation and maintenance of integrated care	++	++	+
Installing a coordinator working at chain-care level	+	++	
Reaching agreements among care partners on tasks, responsibilities and authorizations	+	++	
Developing a multi-disciplinary care-pathway	+	+	
Offering case management for clients with complex needs	+	+	
Defining the ambitions and aims of the collaboration in the care chain	+	++	
Installing improvement teams at care chain level	+		
Guiding the care chain by emphasizing a collaborative commitment	+		
Achieving adjustments among care partners by means of direct contact		++	
Creating an open environment that encourages experiments and pilot projects		++	
Using a systematic procedure for the evaluation of agreements, approaches and results		+	
Stimulating a learning culture and continuous improvement in the care chain		+	

+: Element is named ≥ 3 times.

++: Element is named ≥5 times.

Empty cell: element is not named or <3 times.

As an additional analyses we compared the self-assessed scores of the participants with the calculation methods used to estimate the phase of development (see methods). The results are presented in Table 5. For all of the methods, the Kappa scores are less than 0.20, which qualifies as poor correlation between self-assessed and calculated phases of development. For the 9 and 10 out of ten rule, no Kappa’s could be calculated because no cases qualified by this method. The findings are similar when comparing self-assessed and calculated phases for each group of integrated care service separately.

**Table 5:** **Self**-**assessed phase versus phase according to calculation methods**

**Method**	**Kappa**	**p-value**	**% Self**-**assessed corresponding with calculated**	**% Self**-**assessed higher than calculated**	**% Self**- **assessed lower than calculated**
6/10	0.106	0.067	32.1	33.3	34.5
7/10	0.118	0.042	33.3	42.8	23.8
8/10	0.094	0.091	31.0	57.1	11.9
9/10	*	*	19.1	77.0	3.5
Impl/40	0.105	0.085	34.5	15.4	50.0

*could not be computed.

## Discussion

### Main conclusions

The results of this study show that the four phases of the Development Model of Integrated Care are confirmed empirically in integrated care practice by the participating integrated care coordinators. To underpin this conclusion we did multiple analyses in a four step approach.

Firstly, respondents confirmed the presented phases and all four phases were chosen by integrated care coordinators for the three patient groups. No phases or important phase characteristics were missed. Secondly, almost all the respondents stated that they had been through the previous phase, illustrating a certain change in development over time. A third result which underpins our conclusion is that elements that were related to earlier phases of development were also implemented earlier in time for stroke and AMI practices. This absence in the case of dementia services could be explained by the fact that they are substantially more recent and started only in 2007, with greater external pressure and time urgency.

Also, we found a relationship between the numbers of implemented and planned elements and the phase of development. In earlier development phases integrated care services had more plans for the future and this number of plans decreases over time. Corresponding with that, services that are in further development phases (in either way of assessment) do have more implemented elements of integrated care. These findings in integrated care services support the empirical validation of the DMIC which was based on the literature and experts in the field of integrated care. Although the phases of the DMIC were confirmed, the developmental process of integrated care services seems not to be linear and predictable. Some respondents mentioned that they were ‘in between’ phases, recognised aspects of two (following) phases or mentioned a fall-back. Phases can overlap or run into each other or there can be a relapse to earlier phases. There are no obvious or strict boundaries between phases. This makes it clear that the phases need to be seen as conceptual presentations, but can be helpful for evaluating and guiding integrated care development. These findings correspond with the related literature about organisational development and life cycles. Multiple authors describe life cycles with four to six phases [6], which was also the result in our study. The recent literature about networks also stresses the non-linear aspects of development, the complexity of collaboration and possibility of obstacles and fall backs [23].

### The general applicability

Although the characteristics of the three groups of integrated care services differ on multiple aspects, the development phases appeal to all of them. This raises the question about the general applicability of the development phases and the question if they are useful for multiple or all kind of integrated care services. In our study, the stroke services can be seen as the ‘oldest’ of the three groups and are also the most developed in terms of number of implemented elements. About two thirds of them are in the third or fourth phase of development. The dementia services’ development is comparable with the AMI services, although the latter have existed for longer. It is remarkable that the dementia services have already experienced such a fast development and implemented such a large number of elements. The recent attention to dementia at client, professional and policy level in the Netherlands, initiatives like the National Dementia Improvement Programme and the development of a method for purchasing integrated dementia care, may have contributed to this. Financial preconditions like integrated budgets are not available for stroke and AMI services. The analyses of phase transitions show that next to CEO and higher management commitment, this condition is seen as the most important factor for proceeding to the next phase. The availability of a coordinator, a multidisciplinary care pathway, case management and clear agreements about roles, tasks, goals and ambitions are, regardless of setting, crucial elements that can speed up or hinder development. The recognition of the phases in all these different services, points out the question if the development phases would also fit in an international context. However we have not studied this, first steps to use the model in a Canadian context are now being undertaken and seem positive. Over the past decade the integration of care has gained increasing attention from managers, health care workers, policy makers and researchers in a large number of countries. There is a worldwide interest to better understand integration, implementing integrated care and stimulating development, regardless of systems or legislation. Whereas the Development Model of Integrated care is (also) based on the international literature and does not focus on a specific (patient) group, it is an interesting suggestion to further research the applicability in other countries.

### Assessment of development phases

Although representatives of stroke, AMI and dementia services felt able to position their practice in one of the phases, the comparisons with the calculated phases based on the model are interesting. The self-assessed scores overlap for about one third with the calculated phases, which are based on the present elements as indicated by the coordinators themselves. The 7-out-of ten rule seems to fit the best with the self-assessed scores (highest kappa, significant p-value). When the calculations methods are more inflexible (eight out of ten or higher), the number of services that seem to overestimate their development rises, indicating that these rules may be too strict. Reasons for the low scores between self-assessment and calculated scores could be that the self-assessment scores are merely based on the integrated care coordinator, whose ability to assess therefore is an important factor. Coordinators may vary in their ability to assess, have different roles or involvement and their judgement is possibly influenced by multiple factors. Multiple studies from the fields of psychology and auditing show that people’s judgement about current situations are influenced by earlier experiences, perceptions about the history and the future, recent failures or successes and their situation compared to others [24,25]. It is possible that these factors also play a role in this study. Our analyses show that increasing the available time per week for coordinators has a positive effect on the overlap between the coordinators’ and model’s phase assessment, which may be a manifestation of a more complete role. Recommended important next steps are therefore involving more key persons per integrated care service. When doing so, consensus among partners about present or future elements can be analysed and also the presence or absence of consensus about the assessed development phase.

### Study limitations

Our study has some limitations. Although the response rates were high, the number of participants per patient group differed. AMI services were only represented by nine out of the twelve, but this is because the number of hospitals with interventional capacities and therefore the number of services is limited. For stroke and dementia, diagnoses and treatment can be initiated in almost every hospital. Further, the knowledge of the integrated care coordinator representing the integrated care service was important for the quality of the data. To optimise this, a number of respondents also consulted their partners in the care services before completing the questionnaire. To ensure that the right respondents took part, we explained the criteria for participation in personal contact with the respondents or even visited them. Nevertheless, it would be interesting to invite more respondents from each integrated care service to add additional perspectives and calculate consensus scores. In current studies in diabetes care, palliative care and non-congenital brain damage care we are now applying this approach.

### Suggestions for further research and practical implications

We have three suggestions for further research. Firstly, expanding this research to other countries with other (policy) contexts is to be encouraged. We think this is interesting because reducing fragmentation in care and improving integrated care is a major issue in many countries. Winning the international Karolinska /EHMA Research award 2012 encourages us to do so. In further research we encourage inviting more respondents per integrated care service. Further analyses on the difference between self assessed phases and the phases as calculated by the model is suggested. Secondly, we suggest further research on the process of integrated care development. Our study gives insight into the phases of development that can be present in practice. It is interesting to monitor and follow the development in each phase. Possible research topics include the implementation strategies taken and which partners or other circumstances are involved at what time.

Thirdly, we suggest further research into the relationship between the development phases and the delivered results in integrated care. It would be interesting to see if integrated care services in further phases of development do have better outcomes on processes, patient satisfaction, quality of life or disease-specific indicators and costs.

Our study also has a number of practical implications. During the survey study, the respondents pointed out that filling in the questionnaire was experienced as a self-evaluation exercise which gave suggestions for the further improvement of their integrated care. When sending in their data, it was notable that they asked for benchmark results. For integrated care practitioners, coordinators and managers the DMIC with its development phases could be used as a quality management tool for multiple patient groups. In quality management the use of self-assessment models is used for reflecting on current practices, for guiding improvement and for improving performance. An example of these models is the European Foundation for Quality Management model [26]. This frequently and internationally used model also describes (groups of) elements that are important for the effective organisation of care. Empirical research shows a clear relationship between the implementation of these elements (‘enablers’) and performance, both in industry [27,28] and in health care [29,30]. The EFQM model is also used as evaluation and improvement tool. The DMIC could also serve as an assessment and evaluation tool to reflect on integrated care practice and may initiate discussions on how to improve and progress to further phases. The model can provide support for steering on quality and with guiding policy and improvement plans. Hence, the DMIC could be regarded as a quality model for implementation of integrated care. The National Stroke Service Network in the Netherlands has adopted the DMIC as such.

In the Netherlands the DMIC is already being used for evaluative purposes by multiple practices in dementia, stroke, youth, palliative, diabetes, non-congenital brain damage and vulnerable elderly care. To simplify filling in the questionnaire, we made a webbased tool based on the model. Other suggestions for practice are to further develop the model into an audit tool and to facilitate benchmarking for learning from comparable others as already practiced in stroke care. The National Stroke Service network has adopted the DMIC as a basis for an audit- and improvement tool for all her members. Health care insurers are also interested and are currently exploring the opportunities to use the model when purchasing integrated care. This year, in cooperation with an insurance company, the self-evaluation of 38 integrated diabetes practices is planned.

## Conclusions

Our study shows that the Development Model for Integrated Care could provide a basis for the development of practice of integrated care. Although the 84 participating integrated care services differed on multiple aspects and patient groups, the four development phases of the DMIC are recognised and confirmed in practice. Objectively self-assessing development phases would appear to be complex. The model can provide support in assessing development phases and giving suggestions for further development. The study suggests that the development of integrated care is a long-term non-linear process, with multiple phases in which different elements of integrated care are relevant. Integrated care coordinators find the DMIC helpful for evaluating their integrated care services and guiding further development. The four-phase model has the potential to serve as a generic quality management tool for integrated care and as a framework for further research on integrated care services and their development.

## Competing interests

The authors declare that they have no competing interests.

## Authors’ contributions

All authors contributed to the study. MM, KA and RH designed the questionnaire on the basis of previous research. MM and RV collected and analyzed the data, supervised by KA and RH. MM led the writing process, while all authors commented on the sequential drafts before approving the final version of the manuscript. All authors have read and approved the final manuscript.

## Pre-publication history

The pre-publication history for this paper can be accessed here:

http://www.biomedcentral.com/1472-6963/13/214/prepub
